# Promotion of Cancer Cell Proliferation by Cleaved and Secreted Luminal Domains of ER Stress Transducer BBF2H7

**DOI:** 10.1371/journal.pone.0125982

**Published:** 2015-05-08

**Authors:** Hideo Iwamoto, Koji Matsuhisa, Atsushi Saito, Soshi Kanemoto, Rie Asada, Kenta Hino, Tomoko Takai, Min Cui, Xiang Cui, Masayuki Kaneko, Koji Arihiro, Kazuhiko Sugiyama, Kaoru Kurisu, Akio Matsubara, Kazunori Imaizumi

**Affiliations:** 1 Department of Biochemistry, Graduate School of Biomedical and Health Sciences, Hiroshima University, Hiroshima, Japan; 2 Department of Anatomical Pathology, Hiroshima University Hospital, Hiroshima, Japan; 3 Department of Clinical Oncology and Neuro-oncology Program, Cancer Treatment Center, Hiroshima University Hospital, Hiroshima, Japan; 4 Department of Neurosurgery, Graduate School of Biomedical and Health Sciences, Hiroshima University, Hiroshima, Japan; 5 Department of Urology, Graduate School of Biomedical and Health Sciences, Hiroshima University, Hiroshima, Japan; Centro Cardiologico Monzino, ITALY

## Abstract

BBF2H7 is an endoplasmic reticulum (ER)-resident transmembrane basic leucine zipper (bZIP) transcription factor that is cleaved at the transmembrane domain by regulated intramembrane proteolysis in response to ER stress. The cleaved cytoplasmic N-terminus containing transcription activation and bZIP domains translocates into the nucleus to promote the expression of target genes. In chondrocytes, the cleaved luminal C-terminus is extracellularly secreted and facilitates proliferation of neighboring cells through activation of Hedgehog signaling. In the present study, we found that *Bbf2h7* expression levels significantly increased by 1.070–2.567-fold in several tumor types including glioblastoma compared with those in respective normal tissues, using the ONCOMINE Cancer Profiling Database. In some Hedgehog ligand-dependent cancer cell lines including glioblastoma U251MG cells, the BBF2H7 C-terminus was secreted from cells into the culture media and promoted cancer cell proliferation through activation of Hedgehog signaling. Knockdown of *Bbf2h7* expression suppressed the proliferation of U251MG cells by downregulating Hedgehog signaling. The impaired cell proliferation and Hedgehog signaling were recovered by addition of BBF2H7 C-terminus to the culture medium of *Bbf2h7*-knockdown U251MG cells. These data suggest that the secreted luminal BBF2H7 C-terminus is involved in Hedgehog ligand-dependent cancer cell proliferation through activation of Hedgehog signaling. Thus, the BBF2H7 C-terminus may be a novel target for the development of anticancer drugs.

## Introduction

The endoplasmic reticulum (ER) is the central cellular organelle responsible for the synthesis, folding and post-translational modifications of proteins destined for the secretory pathway. Various pathophysiological conditions, such as ER calcium depletion, oxidative stress, hypoglycemia, expression of mutant proteins and hypoxia, interfere with the correct folding of proteins, and misfolded or unfolded proteins accumulate in the ER lumen. Such conditions, which are collectively termed ER stress, have the potential to induce cellular damage. The ER responds to these perturbations by activating an integrated signal transduction pathway called the unfolded protein response (UPR) [1,2]. Activation of the UPR leads to transient translational attenuation to decrease the demands made on the ER, transcriptional induction of genes encoding ER-resident chaperones to facilitate protein folding, and ER-associated degradation (ERAD) to degrade the unfolded proteins that have accumulated in the ER. In mammalian cells, there are three well-established ER stress transducers: double-stranded RNA-dependent protein kinase-like endoplasmic reticulum kinase (PERK), inositol-requiring enzyme-1 (IRE1), and activating transcription factor 6 (ATF6) [3–5]. PERK directly phosphorylates the α-subunit of eukaryotic initiation factor (eIF2α), leading to a dramatic reduction in cellular protein synthesis [6]. Conversely, and paradoxically, phosphorylation of eIF2α also upregulates the expression of ATF4 [1]. ATF4 transactivates the expression of the pro-apoptotic protein, C/EBP-homologous protein (CHOP), pro-survival proteins, such as ER chaperones, and anti-oxidative stress proteins [7]. IRE1 processes unspliced forms of *X-box-binding protein-1* (*Xbp1*) mRNA to generate spliced forms of the mRNA [4,8]. XBP1 proteins derived from the spliced forms of *Xbp1* mRNA induce the expression of ER-resident chaperones and ERAD-related molecules. ATF6 is cleaved at its transmembrane domain by site-1 and -2 proteases (S1P and S2P, respectively) in response to ER stress [5,8]. The cleaved ATF6 N-terminus translocates into the nucleus and induces the expression of ER-resident chaperones to facilitate protein folding.

In addition to the three canonical ER stress transducers, there are novel types of ER stress transducers that share domains of high sequence similarity with ATF6. These transcription factors have transcription activation and basic leucine zipper (bZIP) domains in their N-terminus and include BBF2H7/CREB3L2 [9], OASIS/CREB3L1 [10,11], Luman/LZIP/CREB3 [12], CREBH/CREB3L3 [13] and CREB4/AIbZIP/Tisp40/CREB3L4 [14]. One of the OASIS family members, BBF2H7, is preferentially expressed in chondrocytes [15,16]. BBF2H7 is cleaved at the transmembrane domain by regulated intramembrane proteolysis (RIP) in response to ER stress. The cleaved cytoplasmic N-terminus containing the transcription activation and bZIP domains translocates into the nucleus to promote the expression of target genes including *Sec23a* [15]. In contrast, the cleaved C-terminus is extracellularly secreted and directly binds to both Indian hedgehog (Ihh) and its receptor, Patched-1 (Ptch1), followed by activation of Hedgehog (Hh) signaling [16]. The pathway mediated by the BBF2H7 C-terminus plays a role in the proliferation of chondrocytes in developing cartilage. The functions of both the N- and C-terminus are essential for the development of mouse cartilage.

Hh signaling is required for cell differentiation and organ formation during embryogenesis [17]. In the adult, Hh signaling remains active in some organs where it is implicated in the regulation of stem cell maintenance and proliferation. In mammals, the Hh signaling pathway is initiated by three Hh ligands (Sonic hedgehog [Shh], Desert hedgehog [Dhh] and Ihh) [18]. Hh ligands bind to the transmembrane Hh receptor, Ptch1. In the absence of Hh ligands, Ptch1 inhibits the function of the transmembrane protein, Smoothened (Smo), which activates glioma-associated oncogene 1 (Gli1) [19,20]. Once Hh ligands bind to Ptch1, Smo is released from the inhibition and promotes activation of the Gli1 transcription factor. Activated Gli1 initiates the transcription of Hh target genes such as *Gli1*, *Forkhead box l1* (*Foxl1*), *Ptch1*, *Cyclin D1* and *Cyclin E1* [21].

Hh signaling is also involved in cancer promotion. Patients with Gorlin syndrome (or basal cell nevus syndrome) have inherited inactivating mutations in Ptch1, leading to constitutively active Hh signaling in the absence of Hh ligands [22]. These patients have a high incidence of basal cell carcinomas (BCCs), a skin tumor of keratinocytes, in addition to medulloblastomas and rhabdomyosarcomas. Almost all cases of sporadic BCCs are caused by activation of the Hh signaling pathway through Ptch1 loss of heterozygosity and/or inactivating or activating mutations in Smo, which diminish its inhibition by Ptch1. Moreover, several Hh ligand-dependent cancers involving overexpression of Hh ligands have been identified in the past few years, including lung, pancreatic, breast and prostate cancers [23,24]. In all cases, these cancers respond to Hh ligands in an autocrine manner. Hh ligands secreted from cancer cells act on the secreting cells (or neighboring cancer cells) to stimulate cell proliferation and/or survival, leading to tumor growth. Therefore, specific inhibitors of Hh signaling may serve as anticancer agents. However, it remains unclear how Hh ligands, their receptor Ptch1 and the downstream signaling are regulated in cancer cells.

As mentioned above, the BBF2H7 C-terminus activates Hh signaling by directly binding to both Hh ligand and Ptch1, which promotes cell proliferation. Interestingly, the *Bbf2h7* gene was first identified as the partner of *fused in sarcoma* (*FUS*) in a chimeric gene found in low-grade fibromyxoid sarcoma (LGFMS) [25]. Recent reports have shown that Hh and Hh target genes are upregulated in LGFMS patients [25,26]. Therefore, we hypothesized that BBF2H7 may have an important role in the proliferation of Hh ligand-dependent cancer cells. Here, we found that the cleaved BBF2H7 C-terminus is secreted from certain types of cancer cells and promotes cell proliferation through activation of Hh signaling.

## Materials and Methods

### ONCOMINE microarray datasets for *Bbf2h7* expression

Microarray datasets for glioblastoma (The Cancer Genome Atlas—Glioblastoma Multiforme and Brain Lower Grade Glioma DNA Copy Number Data, 2013), invasive ductal breast carcinoma (The Cancer Genome Atlas—Invasive Breast Carcinoma Gene Expression Data, 2011), cervical squamous carcinoma [27], prostate adenocarcinoma [28], colon adenocarcinoma [29], gastric adenocarcinoma (The Cancer Genome Atlas—Stomach Adenocarcinoma DNA Copy Number Data, 2013), and pancreatic adenocarcinoma (The Cancer Genome Atlas—Pancreatic Adenocarcinoma DNA Copy Number Data, 2013) were accessed using the ONCOMINE Cancer Profiling Database (version 4.4.4.4, www.oncomine.org) to investigate *Bbf2h7* expression in various cancers.

### Cell culture

HEK293T (derived from human embryonic kidney; American Type Culture Collection [ATCC]), *Bbf2h7* ^-/-^ mouse embryonic fibroblast (MEF, derived from *Bbf2h7* ^-/-^ mice using previously published protocols [15]), MCF7 (derived from human breast cancer; ATCC) and HeLa (derived from human cervical cancer; ATCC) cells were grown in Dulbecco’s modified Eagle’s medium (Life Technologies) containing 10% fetal bovine serum (FBS). LNCap (derived from human prostate cancer; ATCC) cells were grown in Roswell Park Memorial Institute medium-1640 (Life Technologies) containing 10% FBS. U251MG (derived from human glioblastoma; JCRB Cell Bank) and LS174T (derived from human colon cancer; ATCC) cells were grown in Eagle’s minimal essential medium (DS Pharma) containing 10% FBS. To alleviate ER stress, cancer cells were treated with 4-phenylbutyrate (4-PBA; WAKO), a chemical chaperone. To induce ER stress, cancer cells were treated with 1 μM thapsigargin (TG; WAKO), an inhibitor of ER Ca^2+^-ATPase.

### RNA isolation, reverse transcription-PCR (RT-PCR) and quantitative real-time PCR

Total RNA was isolated by the guanidine phenol method using ISOGEN (NIPPON GENE) according to the manufacturer’s protocol. Aliquots of 1 μg purified RNA were reverse transcribed to first-strand cDNA in a 20 μl reaction volume using 200 U Moloney murine leukemia virus reverse transcriptase (Life Technologies) [30]. PCR was performed using specific primer sets in a total volume of 20 μl consisting of 1 μl cDNA, 0.5 μM of each primer, 0.2 mM dNTPs, 1 U DNA polymerase (Agilent), and PCR buffer (Agilent). The primer sequences were as follows: *Gli1* forward, 5′- GAAGCCGTATGTATGTAAGCTCC-3′; *Gli1* reverse, 5′-CTTGGGCTCCACTGTAGAAATG-3′; *Foxl1* forward, 5′- ACAGGCATAGAGGTGACTTTTGG-3′; *Foxl1* reverse, 5′-TTCACAGAGGCTGAGAGTTTGG-3′; *Xbp1* forward, 5′- ATGGATGCCCTGGTTGCTGAAG-3′; *Xbp1* reverse, 5′- GGGTCCAAGTTGTCCAGAATGC-3′; *β-actin* forward, 5′- TCCTCCCTGGAGAAGAGCTAC-3′; *β-actin* reverse, 5′- TCCTGCTTGCTGATCCACAT-3′. PCR cycle parameters were 20 s at 94°C, 20 s at 58°C, and 25 s at 72°C for 18–33 cycles (*Gli1*, 33; *Foxl1*, 31; *Xbp1*, 25; *β-actin*, 18) followed by 72°C for 5 min. Aliquots (5 μl) of each reaction mixture were electrophoresed on 4.8% polyacrylamide gels. The density of each band was quantified using Photoshop Elements 6.0 (Adobe Systems).

To analyze mRNA expression levels, quantitative real-time PCR was performed using Light Cycler 480 System II (Roche) and Light Cycler SYBR Green I Master (Roche). The PCR reactions were performed using KAPA SYBR FAST qPCR Kit Optimized for Light Cycler 480 (KAPA). All results were normalized to the expression of endogenous *β-actin* mRNA and were expressed as relative increases or decreases in expression compared with the controls.

### Western blotting

Proteins were extracted from cells using cell lysis buffer (1% Triton X-100, 100 mM EDTA, 50 mM NaCl, 10 mM HEPES, 500 mM sucrose, and a protease inhibitor cocktail [MBL International]). The protein concentrations of the cell lysates were determined using a Pierce BCA Protein Assay kit (Thermo). Equal amounts of protein were electrophoresed on 12% SDS-polyacrylamide gels, and then transferred onto polyvinylidene difluoride membranes (Bio-Rad). For immunoblotting, the following antibodies and dilutions were used: polyclonal rabbit anti-BBF2H7 C-terminus (Abcam; 1:1,000), polyclonal mouse anti-BBF2H7 N-terminus (the generated antibody [15]; 1:1,000), monoclonal mouse anti-β-actin (Sigma; 1:3,000), polyclonal rabbit anti-interleukin 6 (IL-6) (Abcam; 1:250), alkaline phosphatase-conjugated anti-rabbit IgG (Sigma; 1:3,000), and alkaline phosphatase-conjugated anti-mouse IgG (Enzo; 1:3,000). Labeled proteins were detected using nitro-blue tetrazolium chloride (WAKO) and 5-bromo-4-chloro-3'-indolylphosphatase p-toluidine salt (Roche).

### Immunoprecipitation

To detect BBF2H7 C-terminus in the culture medium, culture supernatants were incubated with anti-BBF2H7 N-terminus, anti-BBF2H7 C-terminus, or anti-IL-6 antibodies overnight. The samples were then precipitated with protein G agarose beads (Life Technologies), followed by western blotting. IL-6 was used as a loading control.

### Immunohistochemistry

Tumor samples were collected during surgery of Hiroshima University Hospital, frozen in liquid nitrogen and stored at −80°C. Sections were fixed in cold acetone at −20°C and then in a 4% paraformaldehyde phosphate buffer solution. For immunostaining of BBF2H7 C-terminus, we used an anti-BBF2H7 C-terminus antibody as the primary antibody and Dako Envision Kit (Agilent) for the detection according to the manufacturer’s recommendations. All procedures were in accordance with the Ethical Committee for Human Genome Research of Hiroshima University.

### Plasmids and transfection

Human BBF2H7 full-length, N- and C-terminus cDNAs were obtained by blunt-end ligation of PCR products corresponding to amino acids 1–520, 1–377 and 431–520 (GenBank Accession Number, NP_919047.2) with a BiP (binding immunoglobulin protein) signal peptide sequence (amino acids 1–16, GenBank Accession Number, NP_005338.1) at the N-terminus. The BBF2H7 full-length, N- and C-terminus cDNAs were cloned into a pcDNA3.1(+) vector. HEK293T cells and *Bbf2h7* ^-/-^ MEFs were transfected with each plasmid using Lipofectamine 2000 (Life Technologies) according to the manufacturer’s protocol.

### Bioassays of cell proliferation

U251MG cells (1 × 10^5^) were plated on 6-cm dishes, cultured at 37°C for 24 h, and then transfected with scrambled or *Bbf2h7* small interfering RNA (siRNA) using Lipofectamine 2000 according to the manufacturer’s protocol. For *Bbf2h7* knockdown, the following sequences were used: 5′-GGAAGAAGGUAGAGGUUCU-3′ (siBBF2H7-1) and 5′-CCAGAAACCUGCUGAUCUA-3′ (siBBF2H7-2). U251MG cells were incubated at 37°C for 24 h after transfection, and then the culture medium was replaced. Cell numbers were counted at the indicated days after transfection. siRNAs were purchased from Bioneer (Daejeon, South Korea).

Additionally, after transfection with scrambled or *Bbf2h7* siRNAs, *Bbf2h7*-knockdown U251MG cells were exposed to conditioned medium collected from HEK293T cells transfected with an empty vector (Mock) or BBF2H7 C-terminus (C-Sup.), or to C-Sup. in which BBF2H7 C-terminus was pulled down by immunoprecipitation using an anti-BBF2H7 C-terminus antibody (C-Sup. + Ab.). Cell numbers were counted at the indicated days after transfection. For the cell proliferation assay, we also used a cell counting kit (WST-8 assay, DOJINDO) according to the manufacturer’s protocol.

To confirm activation of Hh signaling by BBF2H7 C-terminus, U251MG cells were treated with recombinant Shh (R&D Systems), cyclopamine (WAKO) or purmorphamine (WAKO).

### Statistical Analysis

Statistical comparisons between two samples were made using the Student’s *t*-test. *P* values < 0.05 were considered significant.

## Results

### The expression of BBF2H7 in tumors and secretion of its cleaved C-terminus from cancer cells

**Fig 1:**
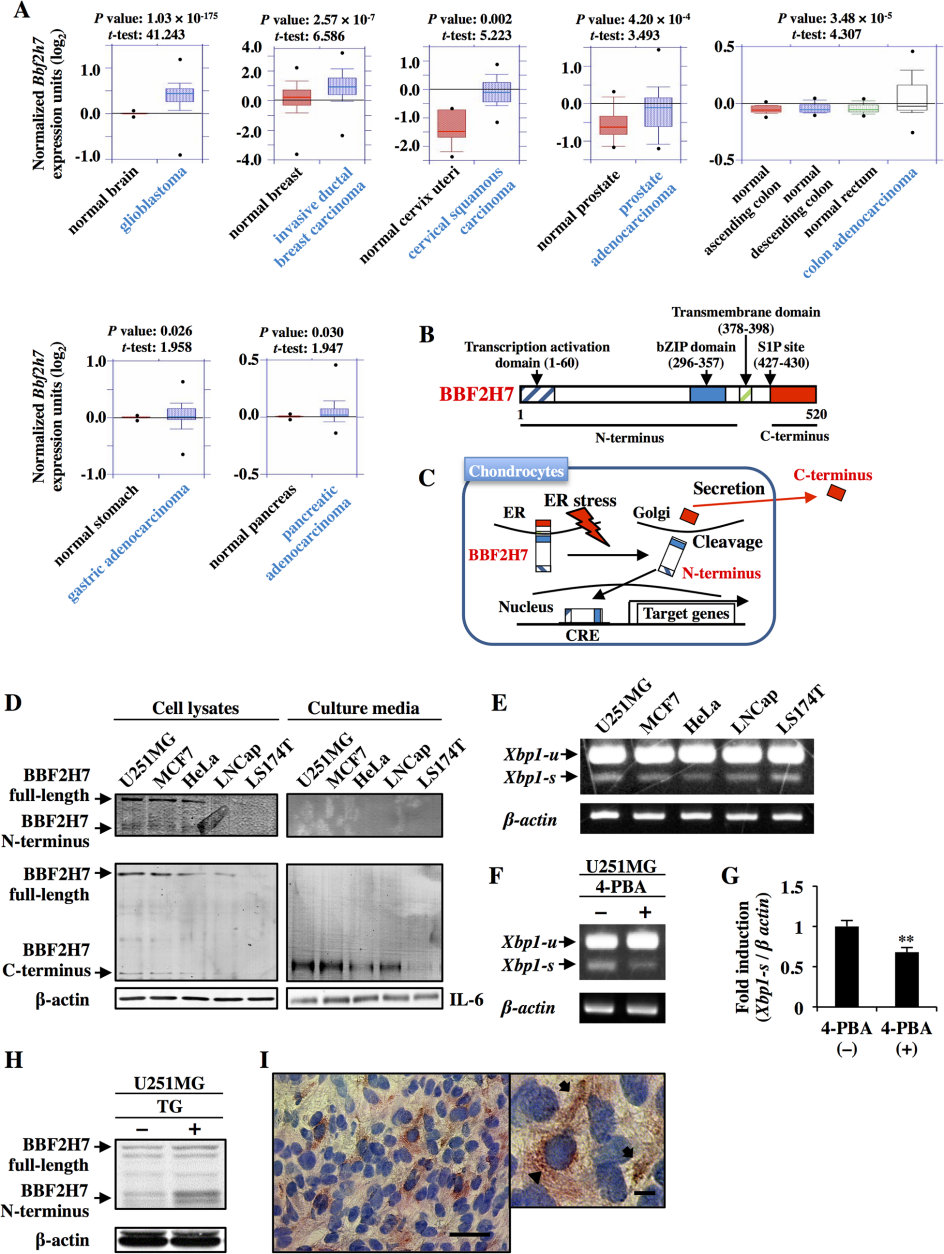
The structure of BBF2H7 and its expression in tumors or cancer cell lines. (A) Increased expression of *Bbf2h7* in human cancers. Microarray datasets of tumors were accessed in the ONCOMINE Cancer Profiling Database (version 4.4.4.4, www.oncomine.org). Box plots showing increased expression of *Bbf2h7* during tumorigenesis of various cancers were constructed from ONCOMINE. The y-axis represents the log_2_ median-centered intensity (normalized expression). The line within the box represents the median expression value for each group, and the upper and lower edges of the box indicate the 75^th^ and 25^th^ percentiles of the distribution, respectively. The lines (whiskers) from each box extend to the 90^th^ and 10^th^ percentiles of the distribution. The black dots outside the ends of the whiskers represent the largest and smallest data points. Box plots depicting the distribution of *Bbf2h7* expression within each sample and a Student’s *t*-test giving a *P* value for the comparison of *Bbf2h7* expression between normal and malignant tissue samples were obtained directly through ONCOMINE. Normal colon tissue samples included ascending colon, descending colon and rectum. (B) Predicted peptide features of human BBF2H7. Transcription activation, basic leucine zipper (bZIP) and transmembrane domains, as well as a site-1 protease (S1P) site are indicated. (C) Under ER stress conditions, BBF2H7 is transported from the ER to the Golgi apparatus and cleaved at the S1P site [9]. The cleaved BBF2H7 N-terminus acts as a transcription factor via binding to the cyclic AMP response element (CRE) of target genes [15]. In contrast, the cleaved BBF2H7 C-terminus is extracellularly secreted [16]. (D) Western blotting of the endogenous full-length BBF2H7 and the N- or C-terminus of BBF2H7 using cell lysates (left panel) or culture media (right panel) of several human cancer cell lines. BBF2H7 N-terminus, BBF2H7 C-terminus or IL-6 in the culture media was immunoprecipitated using anti-BBF2H7 N-terminus (top panel), anti-BBF2H7 C-terminus (middle panel), or anti-IL-6 antibodies (bottom panel), respectively. β-actin or IL-6 was used as loading controls. (E) RT-PCR analysis of *Xbp1* mRNA expression in several human cancer cell lines. *Xbp1-s* and *Xbp1-u* indicate spliced and unspliced forms of *Xbp1*, respectively. Note that *Xbp1-s* was detected in all cancer cell lines, indicating that these cells are undergoing ER stress. (F) RT-PCR analysis of *Xbp1* mRNA expression in U251MG cells treated with 2 mM 4-PBA, a chemical chaperon, for 3 h. (G) Quantification of *Xbp1-s* expression in F. Error bars represent the mean ± SD of five independent experiments. ** *P* < 0.01. (H) Western blotting of BBF2H7 in U251MG cells treated with 1 μM thapsigargin (TG), an inhibitor of ER Ca^2+^-ATPase, for 6 h. (I) Immunohistochemistry of brain sections from glioblastoma patients using an anti-BBF2H7 C-terminus-specific antibody. Strong signals were detected both in the cytosol of the cancer cells (indicated with an arrowhead) and in their surrounding extracellular space (indicated with arrows). Left panel shows a low magnification, and right panel shows a higher magnification of the left panel. Scale bars indicate 50 μm (left panel) and 10 μm (right panel).

The *Bbf2h7* gene was first identified as the partner of *FUS* in a chimeric gene found in LGFMS [25]. Therefore, it is possible that BBF2H7 may be involved in cancer cell proliferation. We examined the expression levels of *Bbf2h7* in various types of tumors using the ONCOMINE Cancer Profiling Database (Fig 1A). The datasets included 37, 61, 5, 28, 94, 94 and 8 normal tissue samples, and 582, 389, 40, 59, 52, 173 and 32 malignant tissue samples of glioblastoma, invasive ductal breast carcinoma, cervical squamous carcinoma, prostate adenocarcinoma, colon adenocarcinoma, gastric adenocarcinoma and pancreatic adenocarcinoma, respectively. As shown in Fig 1A, *Bbf2h7* expression levels showed a significant increase of 1.070–2.567-fold in several tumor types compared with those in their respective normal tissues. In contrast, there was no significant upregulation of *Bbf2h7* expression in gastric or pancreatic adenocarcinomas, indicating that the gene expression of *Bbf2h7* was specifically enhanced in certain tumor types.

BBF2H7 is cleaved at the transmembrane domain in response to ER stress (Fig 1B and 1C) [9]. The N-terminus promotes the expression of target genes including *Sec23a* [15]. In contrast, the C-terminus is secreted from cells and facilitates the proliferation of neighboring cells through activation of Hh signaling in chondrocytes [16]. Next, we examined the expression of full-length BBF2H7 and its cleaved N- and C-terminus in lysates of the following cancer cell lines: glioblastoma U251MG, breast cancer MCF7, cervical cancer HeLa, prostate cancer LNCap and colon cancer LS174T. We detected the 80 kDa full-length BBF2H7 and its cleaved N-terminus (50 kDa) and C-terminus (20 kDa) in the lysates of almost all cancer cell lines except LS174T cells (Fig 1D). BBF2H7 C-terminus, but not full-length BBF2H7 or its N-terminus, was also detected in the culture media of these cell lines, indicating that the cleaved C-terminus was secreted from these cells into the culture media. The cleavage of BBF2H7 depends on ER stress [9]. Hence, we checked whether the cancer cell lines were undergoing ER stress. As shown Fig 1E–1G, the spliced forms of *Xbp1* [8], a marker of ER stress, were observed in all of the cancer cell lines. Treatment of U251MG cells with 4-PBA [31], a chemical chaperone, decreased the spliced forms of *Xbp1* in basal levels and alleviated ER stress, indicating that the cancer cells we checked were under ER stress conditions. Furthermore, we confirmed that BBF2H7 was cleaved in response to ER stress in cancer cells (Fig 1H). These data showed that BBF2H7 was cleaved and its C-terminus was extracellularly secreted in response to ER stress under homeostasis in these cancer cell lines, except for LS174T cells. Next, to confirm that BBF2H7 protein is expressed in tumors, we performed immunohistochemistry using brain sections from glioblastoma patients and an anti-BBF2H7 C-terminus-specific antibody (Fig 1I). Strong signals were detected both in the cytosol of the cancer cells and in their surrounding extracellular space. These results indicated that BBF2H7 was highly expressed in glioblastoma and that the cleaved C-terminus was extracellularly secreted from cancer cells.

### Cancer cell proliferation in an Shh-dependent manner

**Fig 2:**
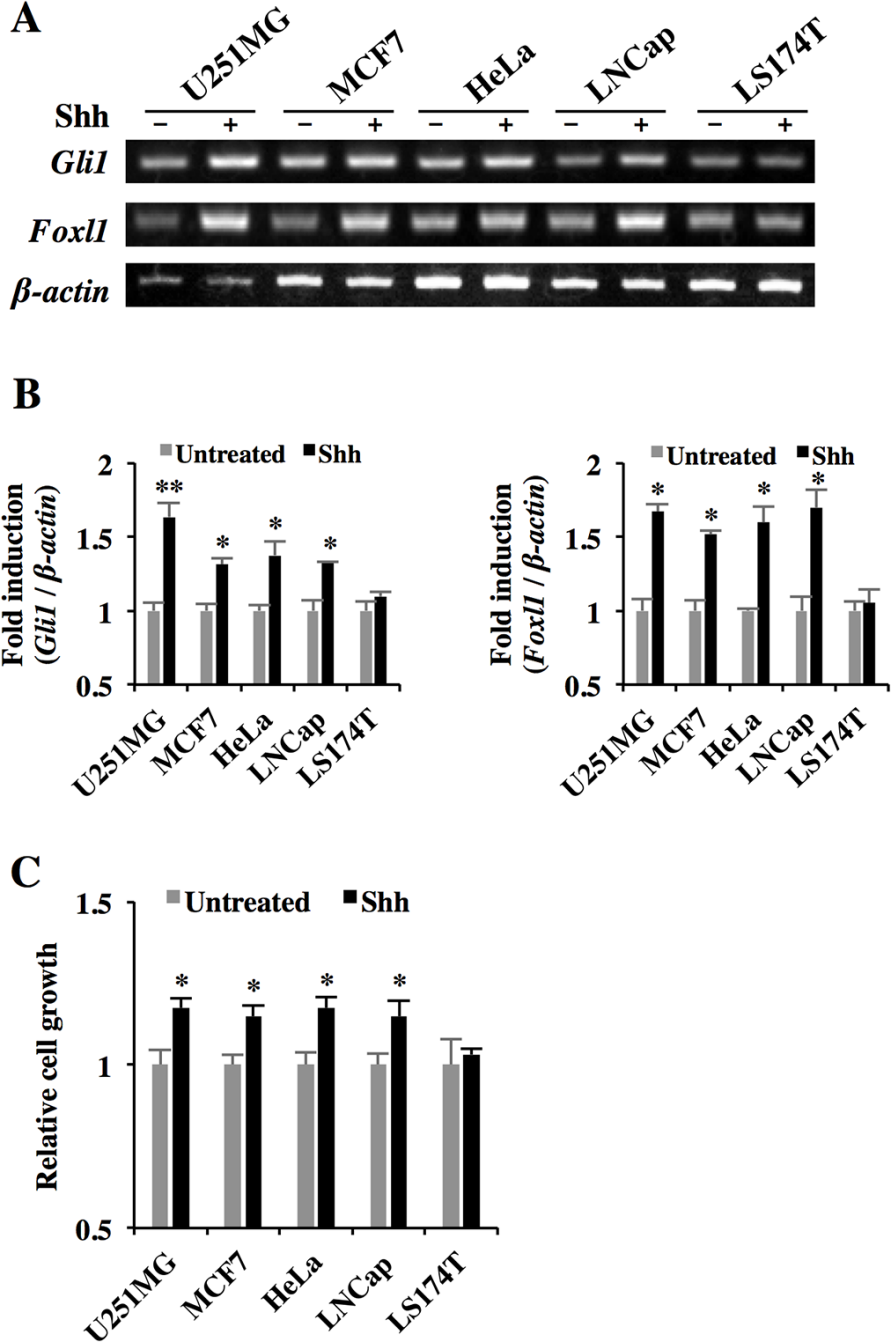
Cancer cell proliferation in an Shh-dependent manner. (A) RT-PCR analysis of Hh target genes in several cancer cell lines treated with 500 ng/ml recombinant Shh for 48 h. (B) Quantitative real-time PCR analyses of Hh target genes expression in A. Error bars represent the mean ± SD of five independent experiments. * *P* < 0.05, ** *P* < 0.01. (C) The cancer cell lines were cultured for 5 days after treatment with 500 ng/ml recombinant Shh and analyzed by WST-8 assays at day 5. Error bars represent the mean ± SD of five independent experiments. * *P* < 0.05.

It is known that BBF2H7 C-terminus binds to both Hh ligand and its receptor, Ptch1, and promotes Hh signaling [16]. To examine whether the cancer cell lines with elevated BBF2H7 expression show enhanced cell growth and activation of the Hh signaling pathway in a ligand-dependent manner, we treated these cancer cell lines with recombinant Shh (Fig 2A–2C). Treatment with recombinant Shh resulted in significant upregulation of Hh target genes such as *Gli1* and *Foxl1* in BBF2H7-expressing cancer cell lines and enhanced cell growth. However, LS174T cells, which did not express BBF2H7, showed no response to treatment with recombinant Shh. These results suggest that the four cancer cell lines that secrete BBF2H7 C-terminus have enhanced cell growth in an Shh-dependent manner.

### BBF2H7 C-terminus enhances Hh signaling in cancer cells

**Fig 3:**
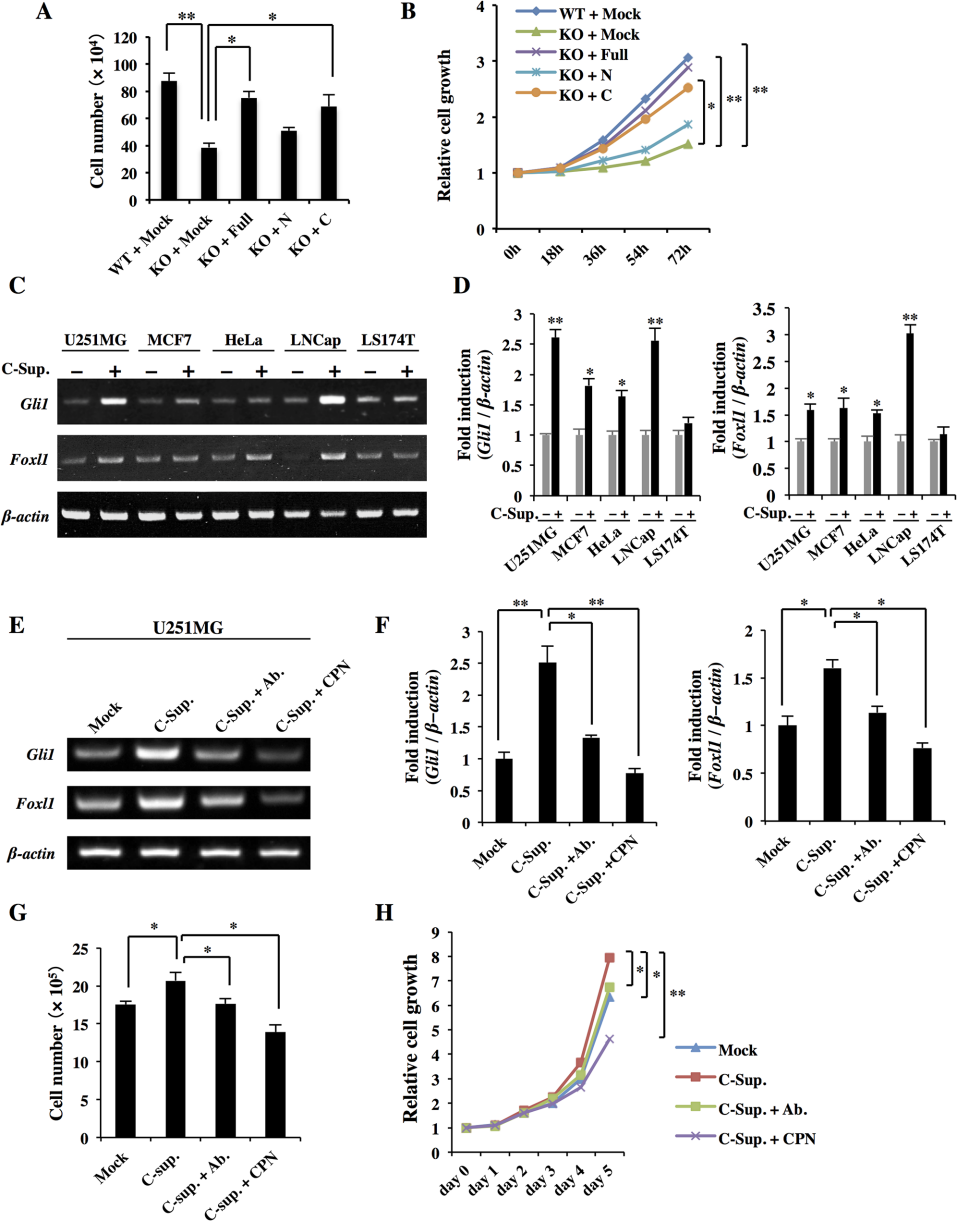
Activation of Hh signaling by BBF2H7 C-terminus. (A, B) *Bbf2h7* ^-/-^ MEFs were cultured for 5 days after transfection with BBF2H7 full-length (Full), N-terminus (N), C-terminus (C) or an empty vector (Mock), and analyzed by cell counting at day 5 (A) and WST-8 assay at the indicated time points (B). WT and KO indicate wild type MEFs and *Bbf2h7* ^-/-^ MEFs, respectively. Error bars represent the mean ± SD of five independent experiments. * *P* < 0.05, ** *P* < 0.01. (C) RT-PCR analysis of Hh target genes in several cancer cell lines exposed to conditioned medium collected from HEK293T cells transfected with BBF2H7 C-terminus (C-Sup. +) or an empty vector (C-Sup.–) for 48 h. (D) Quantitative real-time PCR analyses of Hh target genes expression in C. Error bars represent the mean ± SD of five independent experiments. * *P* < 0.05, ** *P* < 0.01. (E) RT-PCR analysis of Hh target genes in U251MG cells treated with C-Sup., C-Sup. depleted of BBF2H7 C-terminus by immunoprecipitation using an anti-BBF2H7 C-terminus antibody (C-Sup. + Ab.), or C-Sup. in the presence of 5 μM cyclopamine (C-Sup. + CPN). Mock indicates conditioned medium collected from HEK293T cells transfected with an empty vector. (F) Quantitative real-time PCR analyses of Hh target genes expression in E. Error bars represent the mean ± SD of five independent experiments. * *P* < 0.05, ** *P* < 0.01. (G, H) U251MG cells were cultured for 5 days after conditioned medium treatment and analyzed by cell counting at day 5 (G) and WST-8 assays at the indicated days (H). Error bars represent the mean ± SD of five independent experiments. * *P* < 0.05, ** *P* < 0.01.

As shown in Fig 1A and 1D, BBF2H7 was elevated in some tumors and cancer cell lines. We examined the roles of BBF2H7 N-terminus and C-terminus in cell proliferation by transfecting the expression vectors into cancer cells. BBF2H7 N-terminus hardly affected cell proliferation, indicating that the N-terminus does not have the potential to promote cell proliferation in cancer cells. In contrast, BBF2H7 full-length and C-terminus slightly enhanced cell proliferation compared with the control, however, the differences were not significant (data not shown). The production of BBF2H7 full-length and C-terminus by the expression vectors may not be high enough to significantly promote cell proliferation. We think that the produced BBF2H7 full-length and C-terminus by the expression vectors had a smaller effect on cell proliferation, because endogenous BBF2H7 was highly expressed in U251MG cells. Therefore, we transfected human BBF2H7 full-length, N- or C-terminus into *Bbf2h7* ^-/-^ MEFs [15], whose cell proliferation was impaired in comparison to wild type MEFs, to eliminate the effects of endogenous BBF2H7 on cell proliferation (Fig 3A and 3B). Transfection of BBF2H7 full-length or C-terminus into *Bbf2h7* ^-/-^ MEFs restored cell proliferation, however, transfection of BBF2H7 N-terminus alone did not improve cell proliferation, indicating that the human BBF2H7 C-terminus, but not the N-terminus, has the potential to promote cell proliferation.

The secreted BBF2H7 C-terminus has been reported to promote cell proliferation of chondrocytes through activation of Hh signaling [16]. To further investigate the effects of secreted BBF2H7 C-terminus on cancer cell proliferation, the cancer cell lines were exposed to conditioned medium collected from HEK293T cells transfected with human BBF2H7 C-terminus (C-Sup.) containing a BiP signal peptide sequence at its N-terminus, to enable its secretion from cells. Treatment of the cancer cell lines with C-Sup. resulted in significant upregulation of Hh target genes, such as *Gli1* and *Foxl1*, in all the cell lines except for LS174T cells (Fig 3C and 3D). In particular, U251MG and LNCap cells showed more significant induction of Hh target genes, indicating that the effects of C-Sup. on Hh signaling were different among the cancer cell lines. To confirm that the secreted BBF2H7 C-terminus promoted cell proliferation through activation of Hh signaling, we examined whether Hh signaling in U251MG cells would still be activated after depletion of BBF2H7 C-terminus from C-Sup. by immunoprecipitation using an anti-BBF2H7 C-terminus antibody (C-Sup. + Ab.; Fig 3E–3H). As expected, depletion of BBF2H7 C-terminus from C-Sup. caused a significant decrease in the expression of Hh target genes and in cell proliferation. Additionally, treatment with cyclopamine (CPN) [32], a Smo antagonist that inhibits Smo and its downstream Hh signaling, canceled the effects of C-Sup. on the induction of Hh target genes such as *Gli1* and *Foxl1* (Fig 3E and 3F). Moreover, the promotion of cell proliferation by C-Sup. was suppressed by treatment with CPN (Fig 3G and 3H), suggesting that the secreted BBF2H7 C-terminus acts upstream of Smo and enhances Hh ligand-dependent proliferation.

### Effects of *Bbf2h7* siRNA on the proliferation of cancer cells

**Fig 4:**
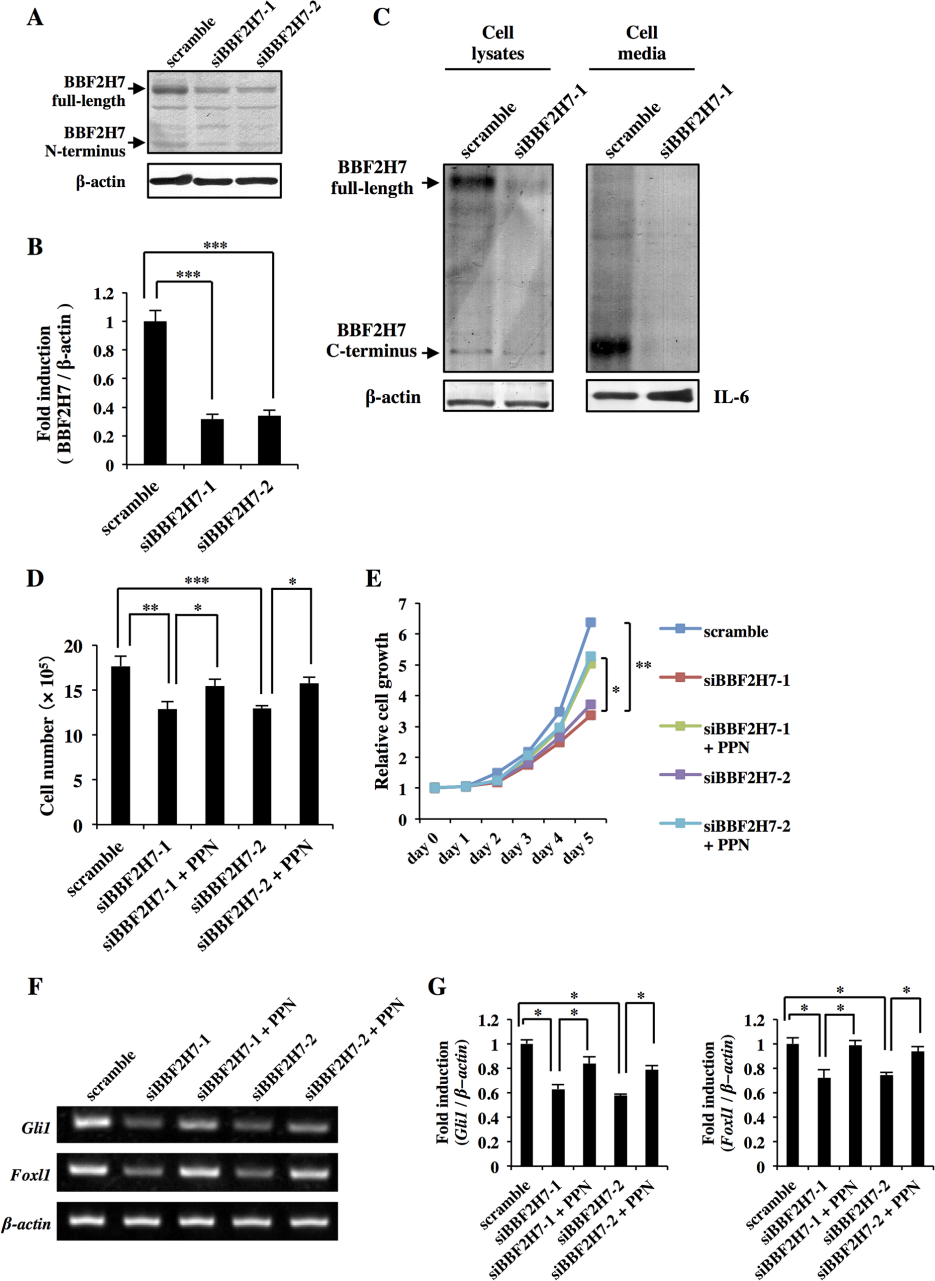
Suppressed proliferation of cancer cells by knockdown of *Bbf2h7*. (A) Western blotting of BBF2H7 in U251MG cells knocked down by siRNAs. Scramble and siBBF2H7 indicate non-targeting siRNA and *Bbf2h7*-targeting siRNAs, respectively. The sequences of siBBF2H7-1 and siBBF2H7-2 have different nucleotide positions. (B) Quantification of full-length BBF2H7 expression in A. Error bars represent the mean ± SD of five independent experiments. *** *P* < 0.001. (C) Western blotting of full-length BBF2H7 or the C-terminus of BBF2H7 using cell lysates (left panel) or culture media (right panel) of U251MG cells knocked down by siRNAs. BBF2H7 C-terminus or IL-6 in the culture media was immunoprecipitated using anti-BBF2H7 C-terminus (upper panel) or anti-IL-6 antibodies (lower panel), respectively. β-actin or IL-6 was used as loading controls. (D–G) U251MG cells were knocked down by siRNAs, and then cell growth was monitored by cell counting and WST-8 assays. (D, E) U251MG cells were cultured for 5 days after transfection with each siRNA in the absence or presence of 2 μM PPN, and then analyzed by cell counting at day 5 (D) and WST-8 assays at the indicated days (E). Error bars represent the mean ± SD of five independent experiments. * *P* < 0.05, ** *P* < 0.01, *** *P* < 0.001. (F) RT-PCR analysis of Hh target genes in U251MG cells at 5 days after transfection with each siRNA in the absence or presence of 2 μM PPN. (G) Quantitative real-time PCR analyses of Hh target genes expression in F. Error bars represent the mean ± SD of five independent experiments. * *P* < 0.05.

We next determined whether knockdown of *Bbf2h7* suppresses cell growth by downregulating Hh signaling in cancer cells. To this end, we used two different siRNAs (siBBF2H7-1 and siBBF2H7-2). Both siRNAs inhibited the expression of endogenous BBF2H7 in U251MG cells by ~70%, indicating that these siRNAs effectively interfered with the expression of endogenous BBF2H7 (Fig 4A and 4B). We also confirmed that the amount of secreted BBF2H7 C-terminus was significantly decreased in the culture medium of U251MG cells transfected with *Bbf2h7* siRNA (Fig 4C). For cell growth experiments, *Bbf2h7* was knocked down by siRNAs in U251MG cells, and then the cell growth was monitored by cell counting and WST-8 assays (Fig 4D and 4E). Cells transfected with scrambled siRNA showed an exponential cell growth curve. In contrast, the proliferation of cells transfected with either siBBF2H7-1 or siBBF2H7-2 was inhibited by ~40% compared with that of the control at 5 days after transfection. Concurrent with the inhibited cell growth, the expression of Hh target genes such as *Gli1* and *Foxl1* was significantly downregulated by transfection of both *Bbf2h7* siRNAs (Fig 4F and 4G). Additionally, purmorphamine (PPN) [33], a Smo agonist, restored cell proliferation and the expression of Hh target genes in *Bbf2h7*-knockdown U251MG cells (Fig 4D–4G). These results suggest that BBF2H7 plays an indispensable role in the proliferation of U251MG cells through activation of the Hh signaling pathway.

### Recovery of cell growth in *Bbf2h7*-knockdown U251MG cells by secreted BBF2H7 C-terminus

**Fig 5:**
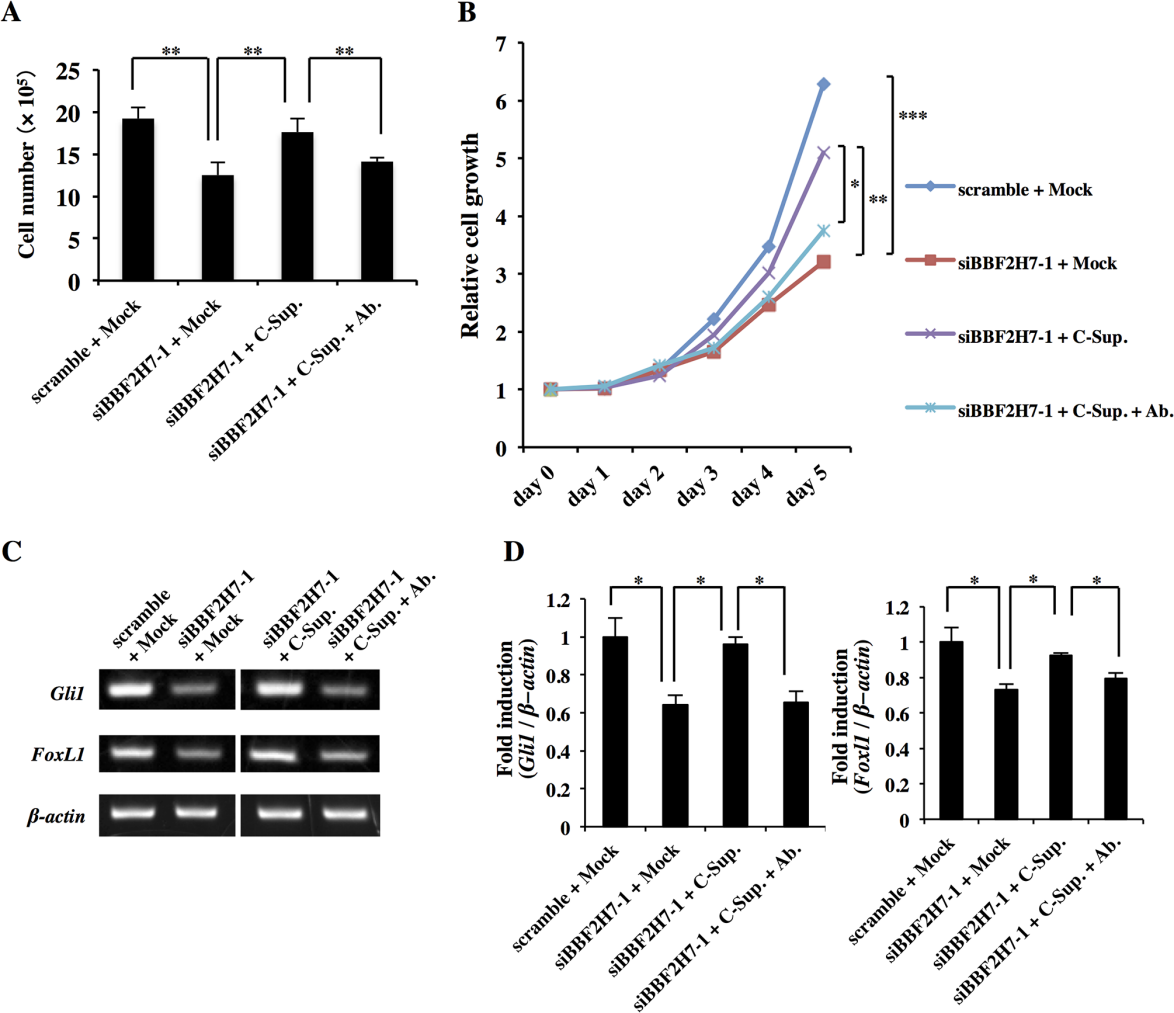
Secreted BBF2H7 C-terminus is involved in the proliferation of cancer cells. U251MG cells were knocked down by siRNAs, followed by treatment with conditioned medium collected from HEK293T cells transfected with an empty vector (Mock) or BBF2H7 C-terminus (C-Sup.), or by treatment with C-Sup. depleted of BBF2H7 C-terminus by immunoprecipitation using an anti-BBF2H7 C-terminus antibody (C-Sup. + Ab.), and then cell proliferation was monitored by cell counting and WST-8 assays. Scramble and siBBF2H7-1 indicate non-targeting siRNA and *Bbf2h7*-targeting siRNA, respectively. (A, B) U251MG cells were cultured for 5 days after conditioned medium treatment, and then analyzed by cell counting at day 5 (A) and WST-8 assays at the indicated days (B). Error bars represent the mean ± SD of five independent experiments. * *P* < 0.05, ** *P* < 0.01, *** *P* < 0.001. (C) RT-PCR analysis of Hh target genes in U251MG cells at 5 days after conditioned medium treatment. (D) Quantitative real-time PCR analyses of Hh target genes expression in C. Error bars represent the mean ± SD of five independent experiments. * *P* < 0.05.

To confirm that the effects of BBF2H7 on the promotion of cell growth are caused by the secreted BBF2H7 C-terminus, *Bbf2h7-*knockdown U251MG cells were exposed to conditioned medium collected from HEK293T cells transfected with the BBF2H7 C-terminus (C-Sup.; Fig 5A and 5B). The growth of *Bbf2h7-*knockdown U251MG cells treated with C-Sup. was restored to that of the control cells. The proliferative effects of C-Sup. were canceled by depletion of the BBF2H7 C-terminus by immunoprecipitation using an anti-BBF2H7 C-terminus antibody. Furthermore, the expression of Hh target genes in *Bbf2h7-*knockdown U251MG cells was significantly decreased compared with that in the control (Fig 5C and 5D). The inhibited expression of Hh target genes was recovered by treatment with C-Sup. The effects of C-Sup. were canceled by depletion of the BBF2H7 C-terminus from C-Sup. by immunoprecipitation using an anti-BBF2H7 C-terminus antibody.

Collectively, these results showed that BBF2H7 was cleaved in response to ER stress, and the cleaved C-terminus was extracellularly secreted from U251MG cells, resulting in promotion of cancer cell proliferation through activation of the Hh signaling pathway.

## Discussion

In the present study, we demonstrated that BBF2H7 is highly expressed in several cancer cell lines, and its cleaved C-terminus is extracellularly released to activate Hh signaling. Inhibition of *Bbf2h7* expression by siRNA resulted in suppression of cancer cell growth by downregulating Hh signaling, indicating that BBF2H7 plays an indispensable role in the proliferation of the examined Hh ligand-dependent cancer cell lines. The ONCOMINE Cancer Profiling Database showed that *Bbf2h7* expression is significantly upregulated in glioblastoma, invasive ductal breast carcinoma, cervical squamous carcinoma, prostate adenocarcinoma and colon adenocarcinoma, but it is unchanged in gastric and pancreatic adenocarcinomas even though their cell proliferation is known to be promoted by activation of Hh signaling [23,24]. Thus, it is conceivable that BBF2H7 is involved in the growth of certain Hh ligand-dependent cancers. Our previous study has demonstrated that the secreted BBF2H7 C-terminus could not activate Hh signaling by itself, but it could bind to both Hh ligand and Ptch1 to facilitate ligand-receptor complex formation, resulting in activation of Hh signaling [16]. Therefore, BBF2H7 C-terminus acts as a co-factor for the ligand-receptor binding and an activator of Hh signaling. Hh/Ptch1-related co-factors such as BBF2H7 C-terminus have been identified, such as growth arrest-specific gene (Gas1), cell adhesion molecule-related/downregulated by oncogenes (Cdon/Cdo), and brother of Cdo (Boc) [34]. These co-factors may modulate cancer cell proliferation in gastric and pancreatic adenocarcinomas in a Hh signaling-dependent manner without significant upregulation of *Bbf2h7*. Co-factors that enhance Hh-Ptch1 binding including BBF2H7 C-terminus, may be good targets for development of novel anticancer drugs that inhibit Hh ligand-dependent growth of cancer cells.

*Bbf2h7* mRNA is highly expressed in certain tumor tissues and cancer cell lines, indicating that *Bbf2h7* expression may be transcriptionally controlled in each cancer cell type. However, the transcriptional regulation of *Bbf2h7* in cancer cells is still unknown. Previously, we have found that the transcription of *Bbf2h7* is regulated by Sex determining region Y-related high mobility group box 9 (Sox9) [35]. The promoter region of *Bbf2h7* contains a Sox DNA-binding motif, and Sox9 promotes the expression of *Bbf2h7* through binding to this element. Recent studies have demonstrated that Sox9 is required for the oncogenesis of several cancer types [36]. Additionally, Sox9 is known to be highly expressed and promote tumorigenicity in prostate cancer, colon cancer and glioblastoma [36]. Interestingly, in glioma cell lines, *Sox9* knockdown by siRNA reduced cell proliferation, implying a certain causal relationship between Sox9 expression and tumor growth [37]. Indeed, *Sox9* expression was increased in the examined cancer cell lines (data not shown). Therefore, it is possible that Sox9 may be involved in the proliferation of cancer cells through induction of *Bbf2h7* expression. However, further investigation is needed to clarify whether Sox9 indeed modulates the expression of *Bbf2h7* in cancer cells.

ER stress is necessary for BBF2H7 cleavage at the transmembrane and secretion of its C-terminus. We detected upregulation of the spliced forms of *Xbp1*, a marker of ER stress [8], confirming that ER stress occurred in the examined cancer cell lines. In many instances, deregulation of ER homeostasis was correlated with the development and growth of cancers [38,39]. Tumor microenvironments can lead to perturbation of ER functions. In particular, hypoxia, nutrient limitation and low pH are known to cause ER stress and activate the UPR in tumors. It is well known that activation of the UPR in tumors enables cancer cells to survive in these unfavorable conditions and promotes tumor growth. Several studies have shown activation of UPR pathways in cancer cell lines [40,41]. It has been well documented that genotoxic insults occur in many cancer cells, and that the cellular responses to destroy damaged cells are often disabled. These physiological changes in the ER environment can adversely affect the folding and maturation of nascent proteins, leading to ER stress, and cleavage of BBF2H7 in response to this ER stress in cancer cells. Therefore, not only inhibition of BBF2H7 C-terminus activities, but also suppression of ER stress, may lead to a novel therapeutic strategy for cancer treatment.

In conclusion, the present study demonstrated that the secreted BBF2H7 C-terminus promoted cell proliferation by activating Hh signaling in certain types of cancer cells. Our results revealed a novel regulatory mechanism of cell proliferation in Hh ligand-dependent cancers, and suggest the potential of BBF2H7 C-terminus as a target for the development of anticancer drugs.
